# Innovative In Vitro Strategy for Assessing Aluminum Bioavailability in Oral Care Cosmetics

**DOI:** 10.3390/ijerph19159362

**Published:** 2022-07-30

**Authors:** Giorgia Allaria, Giulia De Negri Atanasio, Tommaso Filippini, Federica Robino, Lorenzo Dondero, Francesco Soggia, Francesca Rispo, Francesca Tardanico, Sara Ferrando, Stefano Aicardi, Ilaria Demori, Jan Markus, Katia Cortese, Matteo Zanotti-Russo, Elena Grasselli

**Affiliations:** 1 Department of Earth, Environmental, and Life Sciences (DISTAV), University of Genoa, 16132 Genoa, Italy; giorgia.allaria@edu.unige.it (G.A.); lorenzo.dondero@edu.unige.it (L.D.); francesca.rispo@virgilio.it (F.R.); franci24.f@gmail.com (F.T.); sara.ferrando@unige.it (S.F.); stefano.aicardi94@libero.it (S.A.); idemori@unige.it (I.D.); 2 CREAGEN—Environmental, Genetic and Nutritional Epidemiology Research Center, Section of Public Health, Department of Biomedical, Metabolic and Neural Sciences, University of Modena and Reggio Emilia, 41125 Modena, Italy; 3 School of Public Health, University of California Berkeley, Berkeley, CA 94704, USA; 4 Angel Consulting Via San Senatore 14, 20122 Milano, Italy; f.robino@angelconsulting.eu (F.R.); info@angelconsulting.eu (M.Z.-R.); 5 MICAMO S.R.L, 16121 Genoa, Italy; 6 Department of Chemistry and Industrial Chemistry, University of Genoa, Via Dodecaneso 31, 16146 Genoa, Italy; francesco.soggia@unige.it; 7 MatTek In Vitro Life Science Laboratories, 82105 Bratislava, Slovakia; jmarkus@mattek.com; 8 Cellular Electron Microscopy Laboratory, Department of Experimental Medicine (DIMES), Human Anatomy, University of Genoa, 16132 Genoa, Italy; cortesek@unige.it

**Keywords:** aluminum, oral care cosmetics, bioavailability, margin of safety, next-generation risk assessment

## Abstract

Aluminum is an element found in nature and in cosmetic products. It can interfere with the metabolism of other cations, thus inducing gastrointestinal disorder. In cosmetics, aluminum is used in antiperspirants, lipsticks, and toothpastes. The aim of this work is to investigate aluminum bioavailability after accidental oral ingestion derived from the use of a toothpaste containing a greater amount of aluminum hydroxide than advised by the Scientific Committee on Consumer Safety (SCCS). To simulate in vitro toothpaste accidental ingestion, the INFOGEST model was employed, and the amount of aluminum was measured through the ICP-AES analysis. Tissue barrier integrity was analyzed by measuring transepithelial electric resistance, and the tissue architecture was checked through light microscopy. The margin of safety was also calculated. Overall, our results indicate that the acute exposure to aluminum accidentally ingested from toothpastes is safe for the final user, even in amounts higher than SCCS indications.

## 1. Introduction

Aluminum is one of the most abundant elements naturally occurring in the environment as it is the third most common element in the Earth’s crust. For this reason, humans are universally exposed to some forms of aluminum through ingestion, dermal contact, and inhalation [1]. Despite its wide natural distribution, aluminum has no known biological function in humans. In the general population not occupationally exposed to aluminum, the primary source of intake is through diet. After ingestion, less than 1% of aluminum is absorbed through the gastrointestinal system. Its uptake is influenced by several factors including age, pH, stomach contents, type of aluminum compound, and presence of other ligands or ions (e.g., phytates, citrate), thus enhancing or inhibiting its absorption [2].

The contribution of most unprocessed foods is generally limited as the aluminum content is typically low (<5 mg/kg) [3,4,5], although some exceptions worth noting are plants that may accumulate high levels of aluminum, especially tea, leafy vegetables, and some legumes (e.g., soybean) [6,7,8]. Conversely, food processing and packaging tend to markedly increase aluminum content due to the implementation of aluminum-based materials [9]. In particular, aluminum is widely used during cooking for cookware, utensils, as well as for packaging processes in several types of containers (e.g., cans, tubes) and wrappings [10,11]. Moreover, aluminum-containing additives are widely used to enhance food properties (e.g., coloring and anticaking agents) [3,12]. As a consequence, foods most contributing to aluminum intake are generally cereals, vegetables, and beverages (tea and coffee), followed by legumes and sweet products [4,5,6,13,14,15]. The average dietary intake of aluminum is generally well-below the tolerable weekly intake established in 1 mg/kg body weight (bw)/week for the adult European population [3], with a similar evaluation for most aluminum-based additives, having a provisional tolerable weekly intake of 7 mg/kg of bw/week [16]. However, some concerns have been recently raised for a possible dietary intake above the tolerable limits in some vulnerable populations such as infants not exclusively breastfed and young children consuming high amount of aluminum-rich foods [17,18] as well as for some food additives (i.e., sodium and potassium aluminum silicates) [19].

**Figure 1:**
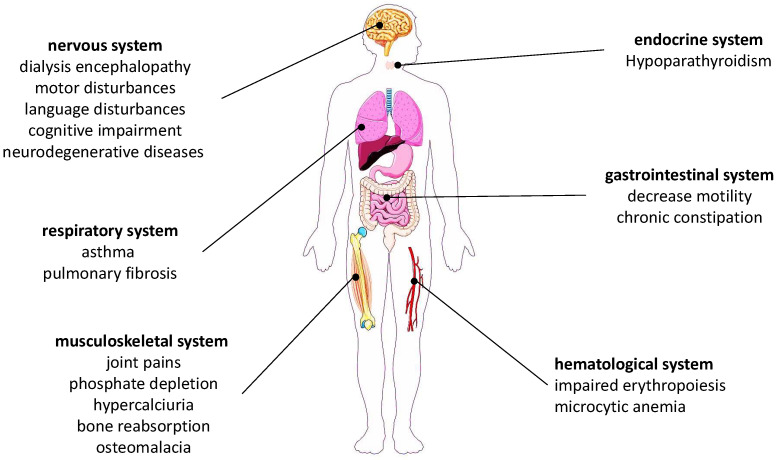
Adverse effects of chronic overexposure to aluminum.

Conversely, chronic aluminum exposure may be of higher concern, especially in people with impaired renal function, as it may cause clinical syndrome of encephalopathy, osteomalacia, and microcytic anemia [20]. Moreover, chronic overexposure to aluminum has been linked to increased risk of neurodegenerative diseases, especially Alzheimer’s disease (Figure 1) [21,22,23,24,25].

Besides the food, another source of intake for aluminum is represented by cosmetic products through dermal contact. In Europe, cosmetics are defined as consumer products intended to be placed in contact with external parts of the human body or with the teeth and the oral cavity with a view exclusively or mainly to cleaning them, perfuming them, changing their appearance, protecting them, keeping them in good condition, or correcting body odors [26]. Due to the extensive use of cosmetics and the absence of a direct advantage for the health, the safety for the customers is the critical point for this class of products and, to preserve consumers’ health, European authorities have defined a strict legal framework based on the safety during use. As a consequence, the European Cosmetic Regulation 1223/2009 requires that each product put on the market undergoes a safety assessment, an evaluation that must be carried out considering a hazard identification; an exposure assessment; a dose–response assessment, including the definition of a Point of Departure (PoD); and a risk characterization [27]. The specific procedure for the assessment of a cosmetic product is based on the evaluation of the single ingredients of the formulation and is described in guidelines published by the Scientific Committee on Consumer Safety (SCCS), the 11th edition [28]. European authorities created a database collecting the substances known to be used as cosmetic ingredient and, at present, this European Commission database for information on cosmetic substances and ingredients lists 76 aluminum derivatives. Depending on their physicochemical properties, these chemicals are used for different purposes in different types of products. It is possible to distinguish the following:

- Aluminum salts, as chlorohydrates, used as antiperspirant: they form an insoluble aluminum hydroxide gel that physically plugs the sweat ducts, reducing the amount of sweat reaching the skin.
- Lakes colorants: complex molecular structures where the colorant is prepared by reacting it with aluminum oxide in aqueous conditions in order to make it insoluble; they are used in make-up products such as lipsticks.
- Insoluble minerals: mainly used in toothpastes as mild abrasive and to improve rheology.
- As part of natural mineral substances used as colorant or pearl in colored cosmetics products such as eyeshadow, face powders, and nail polish.

Aluminum itself is not regulated by EU Cosmetic Regulation 1223/2009; however, we can find restrictions for some derivatives, in particular:

- Aluminum fluoride in oral products for fluorine content (Annex III/34);
- Aluminum zirconium chloride hydroxide complexes AlxZr (OH)yClz and the aluminum zirconium chloride hydroxide glycine complexes in antiperspirant for zirconium and anhydrous aluminum zirconium chloride hydroxide content (Annex III/50);
- Trisodium 5-hydroxy-1-(4-sulphophenyl)-4-(4-sulphophenylazo)pyrazole-3-carboxylate and aluminum lake (CI 19140), Benzenemethanaminium, N-ethyl-N-[4-[[4-[ethyl-[(3-sulfophenyl)-methyl]-amino]-phenyl](2-sulfophenyl)methylene]-2,5-cyclohexadien-1-ylidene]-3-sulfo, inner salts, disodium salt and its ammonium and aluminum salts (Acid Blue Acid Blue 9 Ammonium salt; CI 42090), and Trisodium 1-(1-naphthylazo)-2-hydroxynaphthalene-4′,6,8-trisulphonate and aluminum lake (Cl 16255) when used as hair dye substance in nonoxidative hair dye products (Annexes III/189, III/190, III/192).

It is interesting to note that, so far, European authorities have not considered to restrict by law the presence of aluminum derivatives in cosmetics. However, following the high concern due to clinical data on chronic exposure, the Scientific Committee on Consumer Safety published different opinions on the safe use of these derivatives with the following conclusions:

- when “exposure from non-cosmetic sources of aluminum (food and pharmaceuticals) was aggregated with exposure from cosmetics, food contributed in a similar order of magnitude as cosmetics” [29];
- The use of aluminum compounds is safe up to the following equivalent aluminum concentrations: deo-roll on gel max. 6.18%, Deo Spray antiperspirant max. 3.24%, Deo Spray pump max. 3.24%, Toothpaste max. 3.18%, lipstick max. 14.62%.

In this work, we investigate a tailored testing strategy useful to better estimate the bioavailability of aluminum after the use of a toothpaste containing a greater amount than advised by SCCS (3.18% vs. 38%) of aluminum hydroxide. In fact, the evaluation of a precise systemic exposure is fundamental to refine the safety evaluation of this ingredient and, as a consequence, of the use of the toothpaste itself.

## 2. Materials and Methods

### 2.1. Chemicals

All chemicals were purchased by Sigma Aldrich (Milan, Italy). Toothpaste containing 38% Al(OH)_3_ was obtained from Ludovico Martelli s.r.l., and EpiIntestinal was obtained from MatTeK IVLSL (Bratislava, Slovakia).

### 2.2. In Vitro Digestion According to INFOGEST Model

To assess aluminum leaching in the case of accidental ingestion, toothpastes were subjected to in vitro digestion according to INFOGEST model with slight modifications [30]. Briefly, oral phase was simulated by incubating toothpaste (10 gr) diluted 1:1 (wt/wt) with simulated salivary fluid (15.1 mM KCl; 3.7 mM KH_2_PO_4_; 13.6 mM NaHCO_3_; 0.15 mM MgCl_2_(H_2_O)_6_; 0.06 mM (NH_4_)_2_CO_3_; 1.1 mM HCl; 1.5 mM CaCl_2_(H_2_O)_2_) and incubated for 2 min at pH 7.0 and 37 °C under agitation. The oral bolus was then diluted 1:1 (vol/vol) with simulated gastric fluid (6.9 mM KCl; 0.9 mM KH_2_PO_4_; 25 mM NaHCO_3_; 47.2 mM NaCl; 0.12 mM MgCl_2_(H_2_O)_6_; 0.5 mM (NH_4_)_2_CO_3_; 15.6 mM HCl; 0.15 mM CaCl_2_(H_2_O)_2_), the pH was adjusted to 3.0, and the mixture was incubated for 2 h at 37 °C under agitation. As last step, the gastric digesta was diluted 1:1 (vol/vol) with simulated intestinal fluid (6.8 mM KCl; 0.8 mM KH_2_PO_4_; 85 mM NaHCO_3_; 38.4 mM NaCl; 0.33 mM MgCl_2_(H_2_O)_6_; 8.4 mM HCl; 0.6 mM CaCl_2_(H_2_O)_2_), pH was adjusted to 7.0, and the mixture was incubated for 2 h at 37 °C under agitation. At the end of each phase (oral, gastric, and intestinal), the mixture was centrifuged to separate the insoluble Al(OH)_3_ from Al^3+^ leached into the simulated fluid and stored at −20 °C for further analyses.

### 2.3. In Vitro Aluminum Permeability across the Small Intestine

The quantification of Al^3+^ bioavailability following accidental ingestion was evaluated by incubating Al^3+^ solutions with EpiIntestinal model. This model incorporates enterocytes, Paneth cell, M cells, tuft cells, and intestinal stem cells into a highly differentiated, polarized epithelium. The human cell-based 3D model is cultured at the air–liquid interface to allow physiological (luminal) exposure conditions. EpiIntestinal recapitulates many aspects of normal intestinal function including barrier, metabolism, inflammatory, and toxicity responses, similar to native human intestinal tissue.

Before starting the experiment, EpiIntestinal was preincubated for 24 h in fresh medium. On the day of the experiment, the tissues were transferred to 24-well plates containing 500 microliters per well of Buffer B (HBSS with addition of 1.98 g glucose and 10 mL of 1M HEPES per liter and pH adjusted to 7.4). Samples were diluted in Buffer A (same composition as Buffer B, but the pH was adjusted to 6.8 to mimic pH of intestinal lumen) and applied onto the apical surface of tissue models to a final volume of 5 mL.

**Figure 2:**
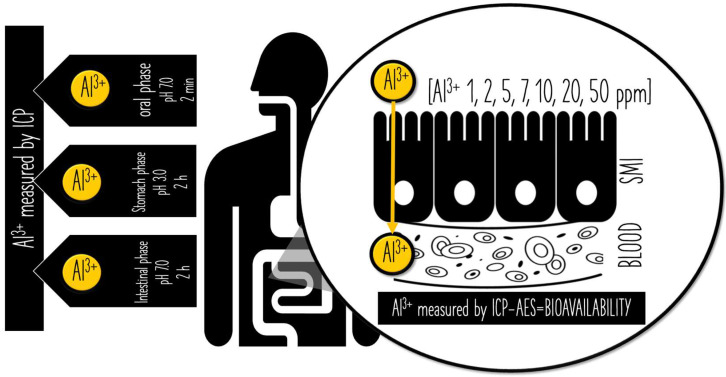
Schematic representation of Al^3+^ leach from toothpaste in a two-step model. The first step consists in INFOGEST COST model composed of oral, gastric, and intestinal phases of digestion. At each phase, the mixture was centrifuged and the supernatant was measured for Al^3+^ leach by ICP-AES. The second step consists in incubating several amounts of Al^3+^ (1,2,5,7,10,20,50 ppm) to assess its passage throughout the small intestine (SMI). The amount of Al^3+^ measured by ICP-AES in the side-B compartment corresponds to the amount of Al^3+^ into the bloodstream (BLOOD) and, ultimately, to Al^3+^ bioavailability.

Treatments were carried out using AlCl_3_ diluted with serial dilution method (1, 2, 5, 10, 20, 50 ppm) starting from AlCl_3_ containing Al^3+^ 980 ppm (evaluated by ICP-AES) as stock solution. The dilution was performed in A-side transport buffer. The inserts were incubated for 2 h at environmental temperature, thus mimicking the duration of intestinal phase. Figure 2 schematizes the coupling of INFOGEST model with EpiIntestinal tissues.

At the end of the experiment, both A-side and B-side buffers were stored at −20 °C for further analyses.

### 2.4. Inductively Coupled Plasma—Atomic Emission Spectroscopy (ICP-AES) Analyses

The samples subjected to the analysis of the aluminum content were mineralized with HNO_3_ s.p. for 50 min at 180 °C, in Easy Prep Plus high-pressure containers (1500 psi, with 1600 W power) using a MARS-5 microwave digestion system (CEM, Matthews, NC, USA).

A Varian (Springvale, Australia) Vista PRO ICP-AES with a sample introduction system consisting of a glass concentric K-style pneumatic nebulizer jointed to a glass cyclonic spray chamber was employed for aluminum determination. All samples were analyzed at the wavelengths of aluminum (308.215 nm, 394.401 nm, and 396.152 nm), using on-line internal standardization (4 mg/L Lu standard solution).

The instrumental detection limit was 0.002 mg/L. The procedural detection limit, evaluated as three times the standard deviation of seven blank samples, was 0.020 mg/L.

Operating conditions of the ICP-AES analysis: ICP RF Power, 1100 W; Plasma gas flow rate, 15.0 L/min; Auxiliary gas flow rate, 1.5 L/min; Nebulizer gas flow rate, 0.75 L/min; Sample uptake rate, 0.78 mL/min; Internal standard (Lu 4 ppm) uptake rate, 0.22 mL/min; Sample uptake delay, 45 s; Stabilization delay, 30 s; Integration time, 15 s; Replicates, 7; Background correction, Fitted; Selected wavelengths (nm), Al (308.215, 394.401, and 396.152), Lu (291.139); Rinse time, 40 s.

### 2.5. Transepithelial Electric Resistance (TEER)

**Figure 3:**
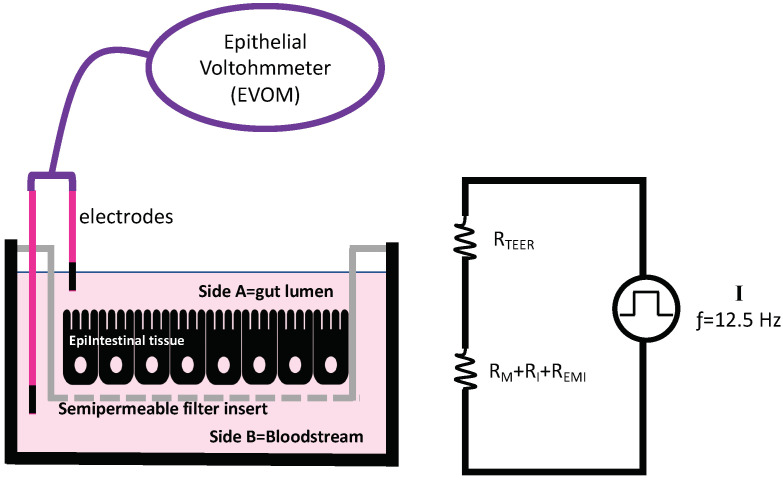
Transepithelial electric resistance (TEER) measurement for assessing EpiIntestinal barrier integrity with Epithelial Voltohmmeter (EVOM). Diagram of electric circuit is also depicted. R_TEER_ [Ω] = total electrical resistance of tissue; RM = resistance of buffer; R_I_ = resistance of semipermeable filter insert; R_EMI_ = resistance of electrode medium interface.

At the end of the bioavailability experiments, EpiIntestinal tissue barrier integrity was assessed in KCl 100 mM by measuring transepithelial electric resistance (TEER) with an Epithelial Voltohmmeter (EVOM; World Precision Machines; Figure 3). The EVOM is able to alternate current voltage signal with a square waveform at a frequency of 12.5 Hz [31].

The two electrodes were placed at the luminal (**A**) and bloodstream (**B**) sides of the insert. The resistance measure of the semipermeable membrane filter alone (without tissue) served as blank (R_BLANK_) and was subtracted from the measure of the resistance across the tissue together with the semipermeable membrane (R_TOTAL_). Tissue resistance was then calculated following the Formula (1)

R_TISSUE_[Ω] = R_TOTAL_ − R_BLANK_ (1)

TEER values were usually reported by normalizing for the surface (insert surface corresponding to 0.6 cm^2^) in units of Ω ×cm^2^ and calculated as [32,33]:

TEER_REPORTED_ [Ω ×cm^2^] = R_TISSUE_[Ω] × S_AREA_ (cm^2^) (2)

### 2.6. Histological Analyses

Each EpiIntestinal insert was washed in phosphate buffered saline (PBS 0.1 M, pH 7.4) and fixed for 1 h in buffered 4% paraformaldehyde (PAF). To be sure that the fixative reached immediately all the cells, the PAF was also gently poured on the insert. After fixation, the inserts were washed again in PBS at 4 °C; dehydrated in ethanol series; cleared in Bioclear (Bio-Optica, Milan, Italy); and finally, embedded in paraffin. The samples were cut in 5 µm-thick sections and put on glass slides. Sections were stained with hematoxylin-eosin histological staining or Alcian-Periodic Acid Schiff (PAS) histochemical staining (Bio-Optica, Milan, Italy) and observed through a light microscope Leica DMRB (Leica Microsystems, Wetzlar, Germany) equipped with a Moticam 3+ (Motic Europe, Barcelona, Spain).

### 2.7. Safety Assessment including Margin of Safety Calculation

For the hazard identification, we considered the impact of aluminum in a developmental toxicity study targeted on the investigation of neurobehavioral endpoints, defining a no observed adverse effect level (NOAEL) of 30 mg aluminum citrate/kg bw/day. The same dose was selected by the joint FAO/WHO Expert Committee on Food Additives (JECFA) for the risk assessment of food additives and then confirmed by SCCS in the opinion on aluminum in lipstick.

## 3. Results

### 3.1. Aluminum Leach Evaluation throughout Digestive System

The passage throughout the digestive system was obtained by INFOGEST method, including oral phase mimicked by incubating 20 gr toothpaste (corresponding to Al(OH)_3_ 7.6 gr and to Al^3+^ 2.63 gr) in simulated salivary fluid at 37 °C for 2 min. Then, the 5% (1 gr), corresponding to the amount that can be accidentally ingested, was incubated with simulated gastric fluid at 37 °C for 2 h (gastric phase), and finally, with simulated intestinal fluid at 37 °C for 2 h (intestinal phase). At each passage, the mixture was centrifuged to pellet the insoluble Al(OH)_3_, thus stopping the eventual further leach into the simulated fluid. The supernatant was then evaluated by inductively coupled plasma (ICP-AES).

**Figure 4:**
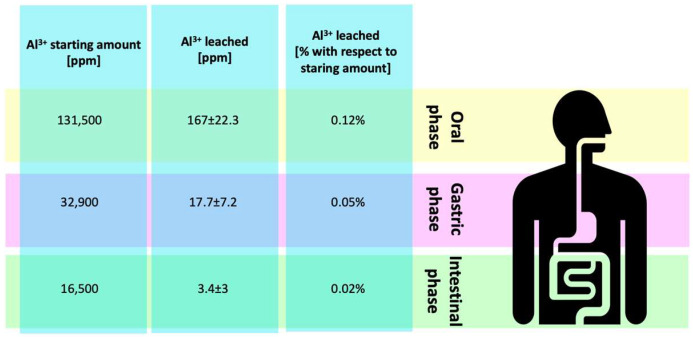
Assessment of aluminum leached from toothpaste during the passage through the digestive tract. Starting amount of Al(OH)_3_ present in the toothpaste is indicated as gr of Al(OH)_3_. Al(OH)_3_ eventually ingested was assumed to be 5%.

Assessment by ICP-AES of Al^3+^ leached in the digestive phases is depicted in Figure 4. A high amount of Al^3+^ leached in salivary simulated fluid (167 ± 22.3 ppm), and the low gastric pH further promoted Al^3+^ release (17.7 ppm corresponding to 0.05%). Finally, the amount released in the intestine was 0.14%, corresponding to about 3 ppm.

### 3.2. Evaluating Bioavailability by Means of Tridimensional Model of the Human Small Intestine

**Figure 5:**
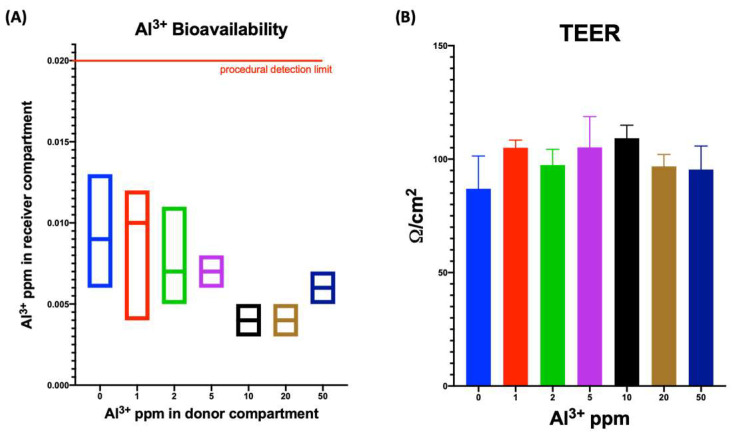
Assessment of Al^3+^ bioavailability corresponding to the passage throughout intestinal mucosa (**A**) and TEER measure (**B**). Measures were performed in triplicate.

A standard solution containing different amounts of Al^3+^ ranging from 1 to 50 ppm was incubated for two hours with the side A of donor compartment corresponding to the luminal side of small intestine (Figure 5A). After incubating for 2 h, the side B buffer, corresponding to the passage into the basolateral side of intestine and thus to the passage into the bloodstream was stored for ICP-AES measures. The Al^3+^ bioavailability was below the detection limit set at 0.02 ppm for all the concentration used on side A of SMI.

At the end of Al^3+^ incubation, tissues were assessed for intestinal mucosa integrity by TEER (Figure 5B). No differences were detected in tissues treated with vehicle (negative control = 0 ppm) compared with all the tissues treated with Al^3+^, thus demonstrating maintenance of the barrier function.

**Figure 6:**
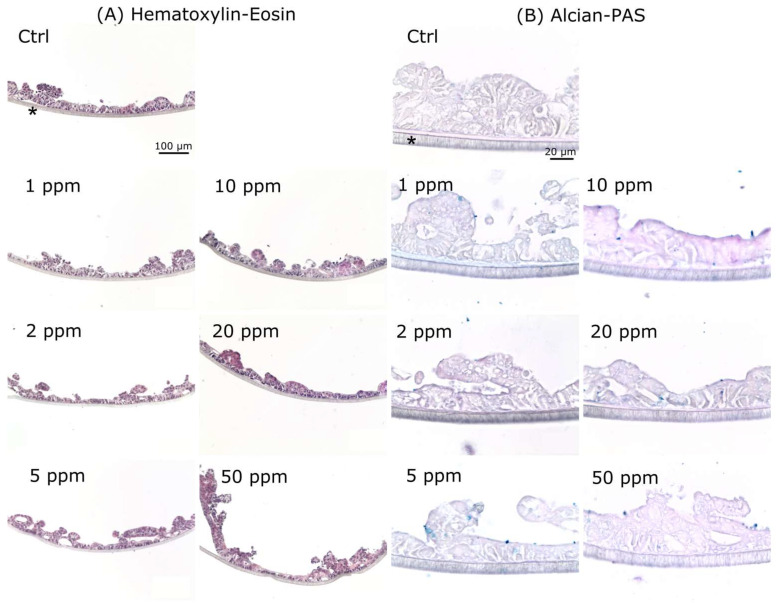
Histological sections of EpiIntestinal inserts exposed to different Al^3+^ treatments. (**A**) Hematoxylin-Eosin staining. The thickness of the insert and the general organization of cells do not show visible alterations compared with the control after Al^3+^ exposure. (**B**) Alcian-PAS staining for mucopolysaccharides. The staining is overall quite weak and does not show any increase in mucopolysaccharide synthesis or accumulation in Al^3+^-exposed inserts compared with the control. Asterisks: microporous membrane (pore size 4 µm) the tissue is cultured on.

Histological evaluation of tissues confirmed that the tested concentrations of Al^3+^ were not able to elicit detectable morphological alterations in the EpiIntestinal insert. The hematoxylin-eosin staining showed an expected morphology of the epithelium and of the short villi, in control and in 20 ppm treatments, as well as in the intermediate concentrations (Figure 6A). The Alcian-PAS histochemistry showed some PAS-positive regions along the tissue (pink) and a small amount of Alcian-positive material (blue) in the control tissue and in all the Al^3+^-treated ones (Figure 6B).

### 3.3. Margin of Safety Calculation

The results of these bioavailability studies show that the absorbed dose corresponded to 0.02 mg of aluminum for each kilogram of sample (0.02 ppm) regardless of the composition of the initial sample.

Therefore, the absorbed amount of aluminum, corresponding to the initial sample of 20 g of toothpaste and to 2.63 g of Al^3+^ (see Table 1), is calculated as 0.0000526 mg (3).

0.02 mg/1000 g = x/2.63 g(3)

Given that the daily exposure for toothpastes suggested by SCCS is 0.14 g, the safety evaluation on 20 g of toothpaste overestimates the amount of product used in daily life.

At this point, it is possible to calculate the relative systemic dermal exposure (SED) by dividing the quantity absorbed by 60 kg, the default body weight valued (4).

SED = (0.0000526 mg/day)/[60 kg] = 0.000000876 mg/kg bw/day(4)

After the definition of the dose–response value (POD) and the relative systemic exposure (SED), it was possible to evaluate the risk correlated to the use of this ingredient calculating the margin of safety (MoS).

In this case, we are considering oral value, and it is possible to define the risk as the ratio of the systemic POD on the relative systemic exposure (SED) (5).

MoS = POD/[SED](5)

For this calculation, we consider as Point of Departure (PoD) the NOAEL value of 30 mg/kg bw/day, the same dose selected by JECFA for the risk assessment of food additives and by SCCS in the opinion on aluminum in lipstick (see Section 2.7).

MoS = (30 mg/kg bw/day)/[0.000000876 mg/kg bw/day] = 34246575.34(6)

The SCCS’s Notes of guidance suggest to consider as safe an ingredient with a MoS over 100—a default value that takes into account corrective factors due to interspecies and intraspecies extrapolation (6).

The calculated values were generously over 100; therefore, it is possible to conclude that the use of aluminum hydroxide at 38% in the investigated toothpaste is safe for the intended use.

## 4. Discussion

In vivo testing, using animal models, has long been used in research to unravel complex biological phenomena occurring through the interaction of several organs, which are, thus, impossible to be investigated using 2D cell culture models. Among these intricate processes, digestion includes several phases occurring in diverse organs. At the end, the digested compounds can be absorbed throughout the intestine or discharged. The physiological process implies both digestion and absorption, thus achieving the transport into the bloodstream of biological components for metabolic processes. This complex phenomenon cannot be merely mimicked by using two-dimensional cell cultures in plastic dishes. Besides, more and more researchers support the application of in vitro models that may satisfy the strategy of 3 Rs, which refers to the Reduction, Refinement, and Replacement of the laboratory use of animals [34], with the obvious advantage of providing simpler and highly reproducible systems where the mechanisms can be studied directly at the cellular level, keeping the translational value of the observed results. Moreover, it is noteworthy that in some cases, such as both finished cosmetic products and substances used as cosmetic ingredients, in vivo testing is prohibited in Europe [35].

In this scenario, the present work describes for the first time the setting of a complex model useful to estimate the bioavailability of aluminum ingested through the use of a toothpaste. Our results offer new insights into the use of tridimensional models coupled with another protocol with the aim of simulating digestion followed by absorption of ingested material of any kind or source, ranging from food to accidentally ingested products, such as toothpaste. Thanks to our approach, it is possible to calculate the Margin of Safety (MoS) for aluminum, considering specific experimental data and not hypothetical considerations. This refinement of the safety evaluation is a first step toward the new approach of the Next-Generation Risk Assessor (NGRA).

**Figure 7:**
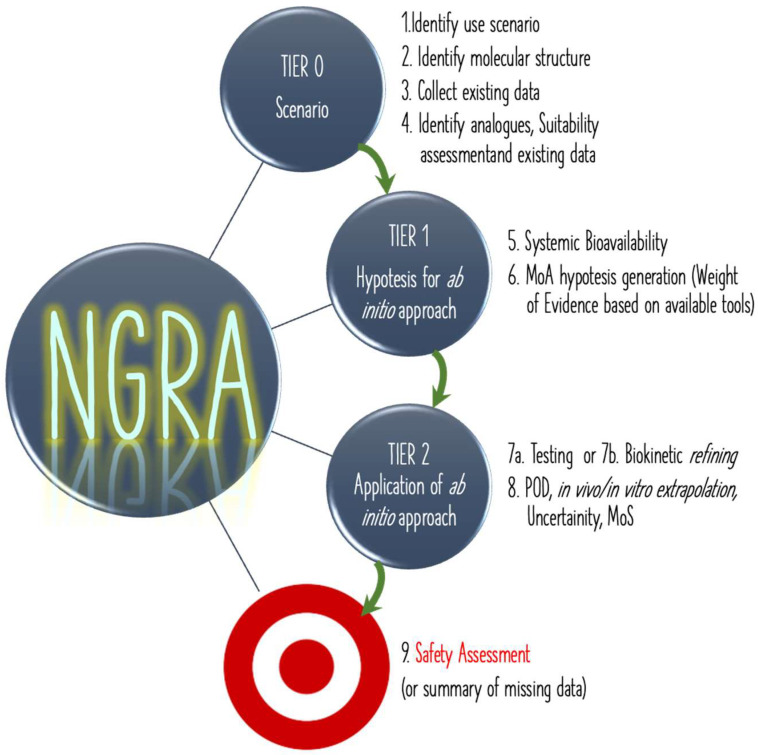
The NGRA frame: tiered, interactive workflow with the aim to perform a safety assessment as final result (red).

NGRA is an evidence-based approach that includes the possibility to take advantage of all currently available data sources, such as read-across information, in silico data, and New Alternative Methodologies (NAM) experimental data, in a systematic strategy useful to conclude a robust risk assessment. This approach overturns the current steps of the risk assessment because all collected general information and specific experimental results must be used to give a conclusion on the initial hypothesis according to a tiered framework (Figure 7) [28].

In this study, we applied the NGRA to the following hypothesis: “is a toothpaste containing 38% of Al(OH)_3_ safe for the final user, even if the amount of aluminum hydroxide is tenfold over the indications of SCCS (3.18 vs. 38%)?” In this regard, with a prudential approach, we chose to consider about tenfold the amount of toothpaste used daily as indicated in the SCCS guideline (2.75 gr vs. 20 gr). The assessment of Al^3+^ present into the bloodstream following toothpaste ingestion was performed by a two-step approach aimed at evaluating as first step the amount of Al^3+^ released by Al(OH)_3,_ corresponding to the soluble fraction. Then, as a second step, we used a tridimensional MatTek model to evaluate the amount of Al^3+^ able to trespass the intestinal mucosa.

We are commonly exposed to aluminum since it is an ubiquitous element. The main sources of aluminum are food; cosmetics; and, to a lesser extent, drinking water [36]. Aluminum absorption occurs through the mucosa of the small intestine [9]. Although the intestinal absorption is generally low, excessive intake may occur due to consumption of a high amount aluminum-rich medicines, leaching from cooking and packaging materials [37], or impairment of renal function. For cosmetics products, aluminum absorption can occur via dermal contact (all kind of cosmetics), inhalation (spray products), and accidental ingestion (lipstick and toothpaste). It is manifest that the bioavailability and retention factor of aluminum are different in these three routes [29]. As a consequence, overexposure to aluminum may alter synthesis and transport of protein, in particular, affecting cytoskeletal pathology, leading to various clinical manifestations and damage of disparate organs and tissues (Figure 1). The mechanism underlying this kind of toxicity is due to the intrinsic nature of Al^3+^ to act as a pro-oxidant by increasing the presence of free radicals. This mechanism has a pleonastic mode of action since aluminum is able to promote lipid peroxidation and also alter signal transduction pathways. In humans, exposure to high levels of aluminum has been linked to adverse effects, especially hematological, bone, and neurodegenerative disorders [38]. Although the absorption of ingested aluminum is very low, some skeletal changes (e.g., osteomalacia) were reported after long-term use of antiacids for the treatment of gastrointestinal disorders. Similarly, dialysis encephalopathy syndrome has been described in subjects after long-term use of intravenous and oral doses of aluminum, due to its accumulation in the brain. Chronic exposure to aluminum, in particular through drinking water, was associated with Alzheimer’s disease [39], although this issue is still highly controversial [40].

The toxicity of a compound is strictly dependent on the dose vehiculated into the bloodstream, and thus, on bioavailability. This parameter is mandatory when assessing a toxic substance. In this regard, a recent work by Priest and colleagues [41] determined the bioavailability of ingested aluminum in a rat model by measuring the amount of Al-labeled aluminum. The data presented for aluminum hydroxide absorption (0.03%) are perfectly in line with our results (0.02%), thus providing further evidence of the validity of in vitro models. Thanks to our integrated approach, we found that acute exposure to a toothpaste, prudentially tested in an excess amount and containing more than tenfold the amount of Al^3+^, results in a widely acceptable MoS value, indicating that the finished product is safe for its intended use. In fact, only a very small fraction of the ingested aluminum was able to trespass the intestinal mucosa.

We conclude that the underlying issue of aluminum exposure through cosmetics is constantly evolving. During the writing of this manuscript, the SCCS’s opinion was updated. The past opinion of the SCCS on aluminum exposure stated that: “although aluminum is also present in cosmetic products, the SCCS considers this source negligible since does not add significantly to the systemic body burden of aluminum” [28]. Recently, a new opinion was published, assessing that when “exposure from non-cosmetic sources of aluminum (food and pharmaceuticals) was aggregated with exposure from cosmetics, food contributed in a similar order of magnitude as cosmetics” [29]. This drastically changes the contribution of cosmetics “to the systemic body burden of aluminum”, thus opening a new point of view towards NGRA approach. No doubt, other studies are also needed that take into account the increased use of aluminum in the food packaging system.

## Figures and Tables

**Table 1:** Stoichiometric ratio for Al(OH)_3_.

Stoichiometric Ratio	Al(OH)_3_	→ ←	Al^3+^	3 OH^−^
Molar weight	78 g/mol		27 g/mol	3 × 17 = 51 g/mol
Weight (gram)	7.6 g		2.63 g	4.97 g

## Data Availability

Not applicable.
